# Supplementary material for: Fucoidan Improves Growth, Digestive Tract Maturation, and Gut Microbiota in Large Yellow Croaker (Larimichthys crocea) Larvae
Source: Nutrients. 2022 Oct 26;14(21):4504. doi: 10.3390/nu14214504 (PMC9654794; doi:10.3390/nu14214504)
Supplement: Supplementary file 1 [file nutrients-14-04504-s001.zip › nutrients-1932615-supplementary.pdf]

nutrients

MDPI

Article

Fucoidan Improves Growth, Digestive Tract Maturation, and Gut Microbiota in Large Yellow Croaker (*Larimichthys crocea*) Larvae

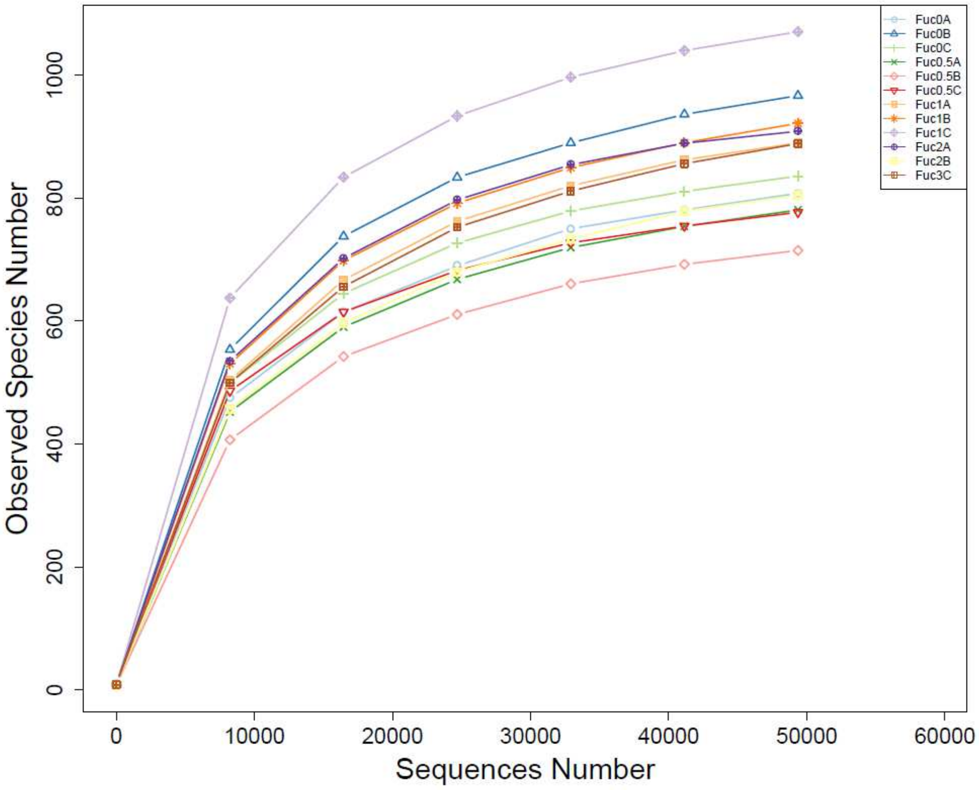

Figure S1. Rarefaction curve of gut microbiota of large yellow croaker larvae (n = 3/group). The rarefied curves for observed species number tended to approach the saturation plateau.

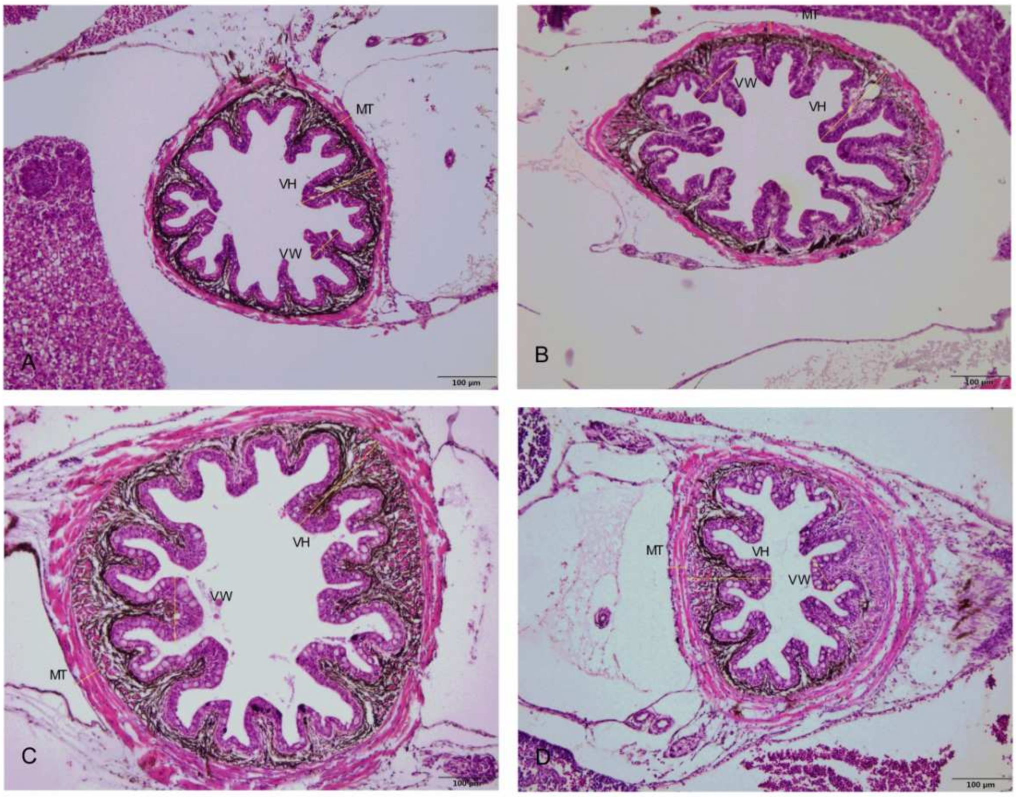

Figure S2. Effects of dietary fucoidan on morphology of the intestine of large yellow croaker larvae (n = 3/group). MT, muscular thickness; VH, villus height; VW, villus width (H&E staining; Scale bar=100 μm; 200 × magnification). (A) Fuc0 group (0.00%); (B) Fuc0.5 group (0.50%); (C) Fuc1 group (1.00%); (D) Fuc2 group (2.00%).

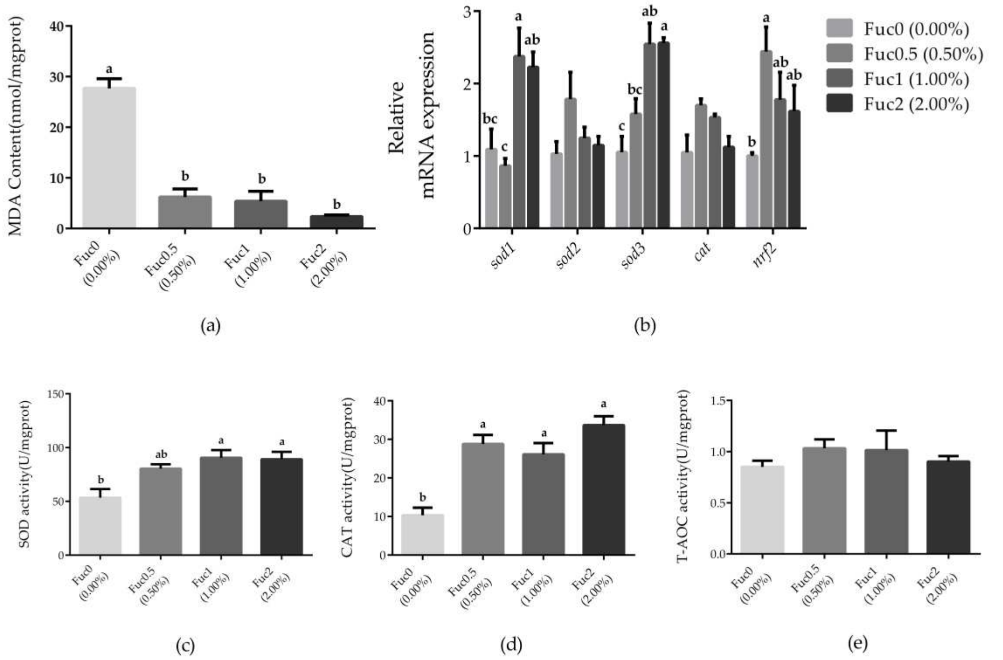

Figure S3. Effects of dietary fucoidan on antioxidant capacity in visceral mass of large yellow croaker larvae (n = 3/group). (**a**) Malondialdehyde (MDA) content; (**b**) antioxidant-related genes

mRNA expression; (**c**) superoxide dismutase (SOD) activity; (**d**) catalase (CAT) activity; (**e**) total antioxidant capacity (T-AOC). Values are means (n = 3), with their standard errors represented by vertical bars. Bars bearing the same letters were not significantly different ($p > 0.05$, Tukey's test).

Table S1. Formulation and proximate analysis of the experimental diets (% dry matter).

Ingredient/% Dry Diet	Experimental Diets (%)			
	**Fuc0 0.00%**	**Fuc0.5 0.50%**	**Fuc1 1.00%**	**Fuc2 2.00%**
White fish meal [1]	34	34	34	34
Krill meal [2]	29	29	29	29
Squid meal [3]	9	9	9	9
Microcrystalline Cellulose	2	1.5	1	0
Yeast extract [1]	2	2	2	2
α-starch	7	7	7	7
Vitamin premix [4]	1.5	1.5	1.5	1.5
Mineral premix [5]	1	1	1	1
Ascorbyl polyphosphate	0.2	0.2	0.2	0.2
Attractant mixture [6]	2	2	2	2
Mould inhibitor	0.05	0.05	0.05	0.05
Antioxidant	0.05	0.05	0.05	0.05
Choline choride	0.2	0.2	0.2	0.2
Fish oil	7	7	7	7
Soybean Lecithin	5	5	5	5
Fucoidan [7]	0	0.5	1	2
Analyzed nutrients composition (dry matter basis %)				
Ash	14.42%	14.55%	14.52%	14.63%
Crude fat	20.42%	20.50%	20.04%	20.31%
Crude protein	52.31%	52.15%	52.82%	52.40%

[1] Commercially available from Guangdong VTR Bio-Tech Co., Ltd. (Zhuhai, China); elementary composition (dry matter): White fish meal, crude protein, 71.73%, crude lipid, 4.76%. [2] Commercially available from Qingdao Bio-ways Ingredients Biotechnology Co., Ltd. (Qingdao, China); elementary composition (dry matter): Krill meal, crude protein, 64.86%, crude lipid, 8.0%. [3] Commercially available from Haixingyuan Feed Co., Ltd. (Hebei, China); elementary composition (dry matter): Squid meal, crude protein, 81.81%, crude lipid, 5.16%. [4] Composition of vitamin premix (IU or mg $^{kg-1}$): vitamin A palmitate, 3000000 IU; vitamin D_3 1200000 IU; DL-α-vitamin E 40.0 mg $^{kg-1}$; menadione, 8.0 mg $^{kg-1}$; thiamine-HCl, 5.0 mg $^{kg-1}$; riboflavin, 5.0 mg $^{kg-1}$; D-calcium pantothenate, 16.0 mg $^{kg-1}$; pyridoxine-HCl, 4.0 mg $^{kg-1}$; inositol, 200.0 mg $^{kg-1}$; biotin, 8.0 mg $^{kg-1}$; folic acid, 1.5 mg $^{kg-1}$; 4-aminobenzoic acid, 5.0 mg $^{kg-1}$; niacin, 20.0 mg $^{kg-1}$; vitamin B_{12}, 0.01 mg $^{kg-1}$; L-ascorgyl-2-monophosphate-Na (3%), 2000.0 mg $^{kg-1}$. [5] Composition of mineral premix (mg $^{kg-1}$ premix): $Ca(H_2PO_4)\cdot H_2O$, 675.0; $CoSO_4\cdot H_2O$, 0.15; $CuSO_4\cdot H_2O$, 5.0; $FeSO_4\cdot 7H_2O$, 50.0; KCl, 0.1; $MnSO_4\cdot 2H_2O$, 101.7; $MnSO_4\cdot 2H_2O$, 18.0; NaCl, 80.0; $NaSeO_3\cdot H_2O$, 0.05; $ZnSO_4\cdot 7H_2O$, 20.0. [6] Commercially available from Qingdao Qihao Biotechnology Co., Ltd. (Qingdao, China). [7] Commercially available from Bright Moon Seaweed Group Co., Ltd. (Qingdao, China).

Table S2. Primers used for quantitative PCR.

Gene	Forward (5'-3')	Reverse (3'-5')	Reference
zo-1 [1]	TGTCAAGTCCCGCAAAAATG	CAACTTGCCCTTTGACCTCT	XM019260744
zo-2 [1]	ACCCGACCTGTTTGTTATTG	ATGCCGTGCTTGCTGTC	[23]
occludin	AGGCTACGGCAACAGTTATG	GTGGGTCCACAAAGCAGTAA	XM010740442
claudin-11	ACCTCCGCCATCAAGCA	TGGGACAAAGAGCCACATC	XM010749420
pcna [1]	AGTTTGCCCGTATCTGCC	CTCTTTGTCTACATTGCTGGTCT	[23]
odc [1]	GAGCCAGGTCGCTTCTATG	CCGTGGTCCCTTCGTCT	[23]
akp [1]	CAGCAGACTCCCGTCCCT	TCCAGTTCGCAGTTCTCATAG	XM019256672
sod1 [1]	GCGGGACCGTGTTCTTTGAG	GCTACCAGCGTTGCCAGTCTTT	[27]
sod2 [1]	GGCACTGGCAAAGGGAGAC	CACAAGCGGCGATACGAAG	[27]
sod3 [1]	CCTGGAGACTTTGGTAACTTTG	TCCCTATTATACTGCTTATGGTG	[27]
cat [1]	CTTCATCAGGGACGCTCTACT	ATGGCATAATCTGGGTTGGT	[27]
nrf2 [1]	TCTGATGTGCGTCTCTCCAG	GCCTCATTCATTTGGTGCTT	[27]
β-actin	GACCTGACAGACTACCTCATG	AGTTGAAGGTGGTCTCGTGGA	[23]

[1] *zo-1*: tight zonula occludens-1; *zo-2*: tight zonula occludens-2; *pcna*: proliferating cell nuclear antigen; *odc*: ornithine decarboxylase; *akp*: alkaline phosphatase; *sod1*: superoxide dismutase 1; *sod2*: superoxide dismutase 2; *sod3*: superoxide dismutase 3; *cat*: catalase; *nrf2*: nuclear factor-E2 related factor 2.

Table S3. Effects of dietary fucoidan on α-diversity index of gut microbiota of large yellow croaker larvae (Means ± S.E.M., n = 3) [1].

Parameters		Experimental Diets (Fuc%)			
		**Fuc0 0.00%**	**Fuc0.5 0.50%**	**Fuc1 1.00%**	**Fuc2 2.00%**
Richness estimates	Observed species	870±49 [ab]	758±21 [b]	961±56 [a]	868±32 [ab]
	Chao1	936.04±51.27 [ab]	821.05±13.98 [b]	1028.88±57.35 [a]	926.66±24.93 [ab]
	ACE [2]	947.02±52.37 [ab]	824.67±20.13 [b]	1043.64±55.30 [a]	951.41±24.62 [ab]
Diversity estimates	PD whole tree [2]	75.48±2.85 [ab]	67.29±2.14 [b]	82.59±3.33 [a]	75.49±2.40 [ab]
	Shannon	5.91±0.06	5.42±0.16	5.88±0.30	5.70±0.24
	Simpson	0.95±0.01	0.93±0.01	0.93±0.02	0.94±0.01

[1] Data in the same row sharing a same superscript letter are not significantly different determined by Tukey's test. [2] ACE: abundance-based coverage estimator; PD whole tree: phylogenetic diversity whole tree.
